# Reciprocal Associations Between Relative or Absolute Physical Activity, Walking Performance, and Autonomy in Outdoor Mobility Among Older Adults: A 4-Year Follow-Up

**DOI:** 10.1177/08982643241282918

**Published:** 2024-09-11

**Authors:** Katja Lindeman, Kaisa Koivunen, Timo Rantalainen, Merja Rantakokko, Erja Portegijs, Taina Rantanen, Laura Karavirta

**Affiliations:** 1 Faculty of Sport and Health Sciences and Gerontology Research Center, 4168 University of Jyväskylä, Jyväskylä, Finland; 2 4169 The Wellbeing services county of Central Finland, Jyväskylä, Finland; 3 University Medical Center Groningen, Center of Human Movement Sciences, 505889 University of Groningen, Groningen, The Netherlands

**Keywords:** mobility, physical activity, physical function

## Abstract

**Objectives:** To examine the reciprocal associations between walking performance, physical activity (PA), and perceived autonomy in outdoor mobility in 322 older adults. **Methods:** At baseline and four years later, a 6-min walk test assessed walking performance. A thigh-mounted accelerometer monitored relative PA (acceleration exceeding the individual’s preferred walking intensity on the walk test) and absolute MVPA (acceleration exceeding 3 METs) in free-living. Autonomy in outdoor mobility was self-reported using the IPA subscale. Cross-lagged panel model was used for analyses. **Results:** Higher relative PA at baseline predicted better walking performance four years later and vice versa (*p* < .05). Baseline MVPA did not predict subsequent walking performance, but better initial walking performance predicted higher subsequent MVPA (*p* < .001). In both models, only walking performance predicted perceived autonomy at follow-up (*p* < .05). **Discussion:** Accumulating enough PA of a sufficient relative intensity can maintain good walking performance, which in turn helps to maintain perceived autonomy in mobility.

## Introduction

In recent decades, the importance of mobility in promoting independent and healthy aging has been increasingly recognized (Satariano et al., 2012), and the ability to be mobile and go outdoors has been identified as crucial for maintaining autonomy in later life (World Health Organization, 2015). Definitions of mobility in aging research have varied, but a consensus has emerged to define mobility as the ability to move with or without assistive devices (Reijnierse et al., 2023). In addition, Beauchamp et al. (2023) recently proposed a framework in which three complementary but distinct facets could be considered when measuring mobility in older adults: locomotor capacity for mobility, actual mobility, and perceived mobility. In the framework, locomotor capacity refers to what a person can do in a test situation and is typically assessed through performance-based measures. Actual mobility refers to what a person does in everyday life and this information can be obtained through self-report or devices, such as accelerometry or GPS. Perceived mobility refers to self-reported measures of what a person believes they can do.

Walking performance is a fundamental aspect of mobility and serves as a cornerstone for engaging in a range of daily activities, both indoors and outdoors. Walking performance, as assessed, for example, by the distance covered in the 6-min walk test, is considered to be the sixth vital sign (Fritz & Lusardi, 2009) and has been shown to be predictive of several health outcomes, including the onset of disability (Perera et al., 2016), cognitive decline (Kikkert et al., 2016), depression (Chan et al., 2020), incident cardiovascular disease, and premature mortality (Veronese et al., 2018). The relationship between walking performance and actual mobility, that is, physical activity (PA), is complex and likely reciprocal. Higher levels of PA are correlated with higher initial physical capacity and less decline over time. On the other hand, lower levels of PA may be due to physical limitations and health problems (Laddu et al., 2020). However, only a limited number of studies have investigated these reciprocal associations. Some have found such associations between PA and physical capacity (Metti et al., 2018), while others have suggested that, for example, walking performance may have a stronger influence on subsequent PA than vice versa (Best et al., 2018).

Perceived mobility could also refer more broadly to perceived autonomy in mobility. Perceived autonomy in outdoor mobility refers to satisfaction with opportunities to access places and participate in activities outside the home, and it is considered optimal when individuals perceive that they have control over decisions about their mobility and the freedom to live according to their preferences (Cardol et al., 2002). For older people, perceived limitations to participation often relate specifically to outdoor mobility (Wilkie et al., 2006) and, for example, existing walking difficulties have been associated with a reduced sense of autonomy in outdoor mobility along with lower levels of PA (Leppä et al., 2021; Skantz et al., 2019). However, while impaired walking performance may affect, it does not necessarily preclude, a sense of autonomy in outdoor mobility (Portegijs et al., 2014). To date, few studies have addressed autonomy in outdoor mobility, and its associations with other dimensions of mobility, such as walking performance and PA, are not yet fully understood.

One study using accelerometers suggested that the reciprocal associations between physical capacity and PA may vary depending on the intensity of PA and the measure used to assess physical capacity (Yerrakalva et al., 2022). While accelerometers provide a method to continuously measure PA in free-living conditions, there is an ongoing debate as to whether absolute or relative (i.e., individualized) thresholds should be used when quantifying daily minutes of PA (Pedišić & Bauman, 2015; Stevens et al., 2020). Although absolute and relative thresholds do not necessarily align, an absolute threshold corresponding to three metabolic equivalents (METs) has been recommended (Garber et al., 2011) and has been widely used to quantify moderate-to-vigorous physical activity (MVPA) in different populations, including older adults (Ekelund et al., 2019; Matthews et al., 2012). However, the choice between absolute and relative thresholds for quantifying accelerometer-based PA in older adults may lead to different conclusions regarding PA, aging, and health (Schrack et al., 2018). Our previous findings have shown that the use of an individual threshold, determined by the acceleration corresponding to an individual’s preferred walking intensity, results in a similar amount of PA regardless of age or sex (Karavirta et al., 2020), providing a promising indicator for investigating the independent role of free-living PA on health and physical function in older adults. To our knowledge, it has not been investigated whether PA, defined in absolute or relative terms, has a different impact on the observed associations between different dimensions of mobility.

Gaining a better understanding of the potential reciprocal and temporal associations between different dimensions of mobility will elucidate how these complementary aspects evolve over time and potentially influence each other as individuals age. A better understanding of these associations will lay the ground for planning public health interventions that target potentially modifiable dimensions of mobility early on, which will be central, for example, to maintaining autonomy in outdoor mobility, one of the key elements of good quality of life. Furthermore, with the increasing use of accelerometers to measure PA in older individuals, it is important to gain a better understanding of how absolute and relative thresholds may influence observed associations with other dimensions of mobility.

Therefore, in this exploratory population-based study, we aimed to apply the framework presented by Beauchamp et al. (2023) and explore longitudinal reciprocal associations between walking performance, PA, and autonomy in outdoor mobility using a cross-lagged panel analysis, which allows for the exploration of autoregressive and cross-lagged pathways in longitudinal data (Selig & Little, 2012). Diverging from Beauchamp et al. (2023), we operationalized perceived mobility more broadly as a perceived autonomy in outdoor mobility. In addition, we aimed to assess whether the use of a threshold scaled to an individual’s preferred walking intensity (relative PA) compared with a fixed threshold for quantifying absolute MVPA differentially influenced the reciprocal associations between walking performance, PA, and autonomy in outdoor mobility.

## Methods

### Study Design and Setting

This study forms part of the data from the “Active aging - resilience and external support as modifiers of the disablement outcome” (AGNES) cohort study. In brief, the AGNES study involves community-dwelling older people living in the city of Jyväskylä in Central Finland. Baseline data were collected between 2017 and 2018, and the initial baseline sample consisted of 1021 participants aged 75, 80, or 85 years old. Comprehensive details of the baseline study protocol and recruitment procedures have been previously published elsewhere (Portegijs et al., 2019; Rantanen et al., 2018). Follow-up data were collected between 2021 and 2022, with a total of 663 participants (Lindeman et al., 2024).

**Figure 1.**
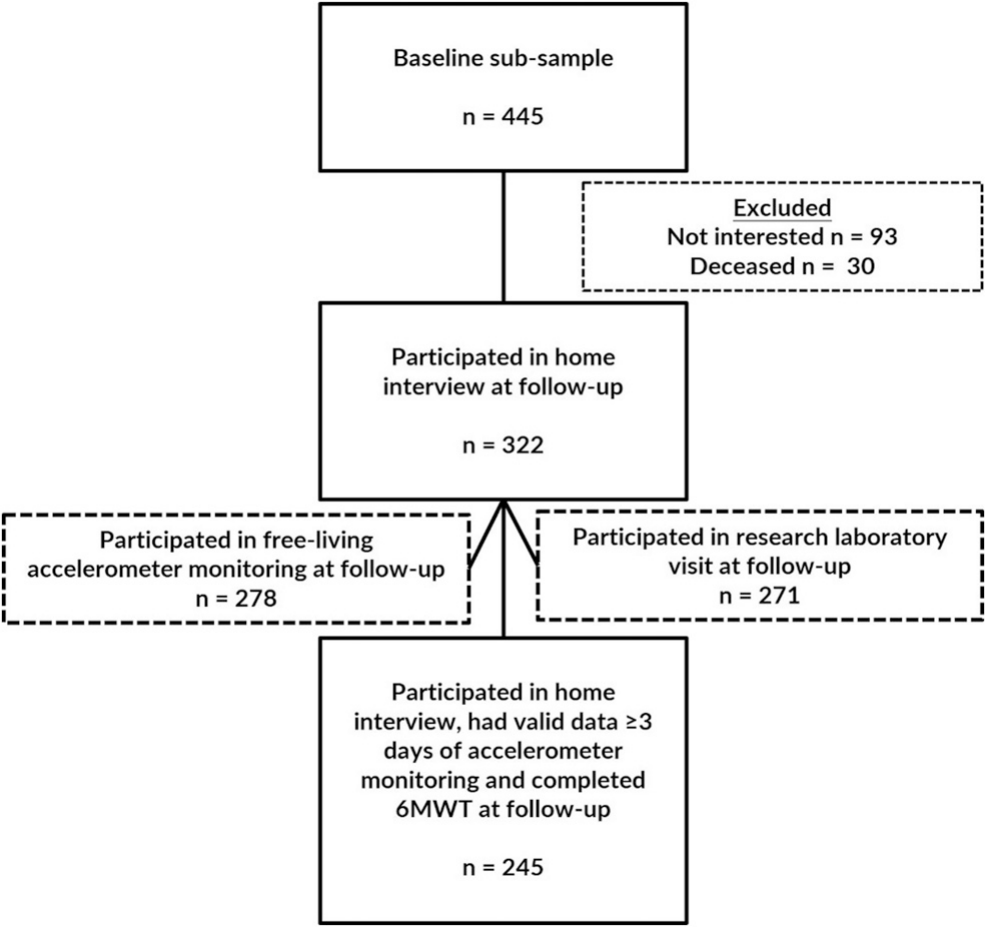
The composition of the study sample.

This study uses data from a baseline sub-sample who participated in free-living accelerometer monitoring and completed a modified 6-min walk test (6MWT) in the research laboratory. At baseline, a total of 445 participants had at least three days of free-living accelerometer data and completed the 6MWT to calculate the daily minutes of relative PA. Of the 445 participants, 322 participated in the home interview at the follow-up in 2021–2022. Of these, 278 people participated in the free-living accelerometer monitoring and 274 of them had valid data for at least three days, while data from four participants were excluded due to technical problems. In addition, 271 participants also attended the follow-up visit at the research laboratory, of whom 260 completed the 6MWT, while 11 participants were not granted medical clearance to attend the test. A total of 245 participants at follow-up had at least 3 days of valid accelerometer data and completed the 6MWT to quantify estimates of daily MVPA and relative PA minutes (Figure 1).

### Ethics

The Ethics Committee of the Wellbeing Services County of Central Finland provided a positive ethical statement for the AGNES baseline study on 23 August 2017 and for the follow-up study on 7 September 2021. The AGNES study follows the principles of the Declaration of Helsinki, and all participants signed an informed consent form before participating in each phase of the study.

## Measurements

### Walking Performance

Walking performance was assessed using a modified 6-min walk test (6MWT) performed at a self-selected usual pace at both time points. The 6MWT was performed in the research laboratory in a 20-m indoor corridor. The total test time of 6 minutes was measured with a stopwatch. Photocells (Faculty of Sport and Health Sciences, University of Jyväskylä, Jyväskylä, Finland) connected to the computer were placed 18.0 m apart and 0.83 m from each end to record lap times. Participants were allowed to stop and rest during the test (but the test time was not stopped for the rest) and to use a walking aid if necessary. Laps were manually calculated during the test and the test was supervised by a trained research assistant. The total distance walked in 6 minutes was measured in meters (m). The 6MWT, administered at a self-selected pace, has been shown to be a feasible measure of walking performance in older adults (Singh et al., 2014), and has demonstrated good construct validity, good test-retest reliability, and responsiveness to change (Holland et al., 2014).

In addition to testing walking performance, the 6MWT was also utilized to determine the acceleration corresponding to the walking intensity. The intensity of the 6MWT was assessed as the median mean amplitude deviation (MAD), which was later used as an individual threshold to quantify relative PA (i.e., daily minutes of PA at or above the preferred walking intensity). MAD (1/n*∑|r_i_ – ř|) was calculated from the vector magnitude (Euclidian norm) of the resultant acceleration (resultant = √X2+Y2+Z2) in non-overlapping 5-s epochs (Vähä-Ypyä et al., 2015). To calculate the median MAD from the 6MWT, the walking test was identified from the accelerometer recording using the accelerometer time stamps in 1-s increments and a record of the test start time. The 6MWT was identified as consecutive epochs of MAD between 0.035 g and 1.2 g lasting for at least 4 minutes and occurring within 20 minutes of the recorded test start time. A minimum of 4 minutes of walking was required out of a total of 6 minutes tested, but a break of a maximum of 1 minute was allowed. The median MAD was calculated as the median of the total walking period, including the potential rest period of up to 1 minute maximum. All algorithmically determined walking periods were visually verified to correspond to the 6MWT (Supplementary Figure 1S).

### Relative and Absolute Physical Activity

Baseline accelerometry data collection has been previously reported in detail elsewhere (Portegijs et al., 2019) and the follow-up data collection was performed according to the baseline accelerometry protocol. Briefly, at both time points, participants wore a triaxial accelerometer (UKK RM42 triaxial accelerometer, continuous sampling at 100 Hz, 13-bit A/D conversion, acceleration range ±16 g, UKK Terveyspalvelut Oy, Tampere, Finland) attached to the mid-thigh of the dominant leg. During the home visit, a research assistant attached the accelerometer to the participant’s thigh with a waterproof film. Participants were asked to wear the accelerometer for seven consecutive days at baseline and five consecutive days at follow-up (24-h wear protocol). The accelerometers were removed by the research assistants when the participants visited to the research laboratory after the monitoring period. Participants also kept simple PA diaries in which they provided information about exercise sessions and the reason and time of any self-removal of the accelerometer. Periods of non-wear were also visually verified from the data, and only days with complete data, that is, 24-h wear from midnight to midnight, were included, and at least three successfully recorded days were required.

The raw accelerometry was calibrated according to previously published protocols (Karavirta et al., 2020; Van Hees et al., 2014). From the resulting gravity-calibrated acceleration values, MAD was calculated as described above. The entire series of 5-s epochs was divided into full 24-h days from midnight to midnight, and finally relative PA and MVPA were calculated for each day. The mean of the included days was used as the outcome measure for each participant.

1. Daily minutes of relative PA were quantified as the accumulated time when intensity ≥ the median MAD of the 6MWT (Karavirta et al., 2020). In contrast to the article Karavirta et al. (2020), this study used the median MAD instead of the mean high-pass filtered vector magnitude, as the median is less sensitive to short rest periods during the test. The individual threshold was calculated separately for the baseline and follow-up.
2. Daily minutes of MVPA were quantified as the accumulated time when intensity ≥ the MAD of 0.175 g, equivalent to 3 METs. The determination of the fixed threshold was based on a treadmill calibration in which older women and men with a mean age of 71.2 years wore the same accelerometer (UKK RM42) at the same site (mid-thigh of the dominant leg). The energy expenditure corresponding to MAD was measured with indirect calorimetry using four different walking speeds from 1.5 to 6.0 km/h. Multiple linear regression with random effects on individual level was used to derive conversion equation for MAD to energy expenditure (Karavirta et al., 2024).

### Perceived Autonomy in Outdoor Mobility

Perceived autonomy in outdoor mobility was assessed using the Impact on Participation and Autonomy (IPA) subscale (Cardol et al., 1999), which was administered as part of the structured face-to-face home interview at both time points. The autonomy outdoors subscale consists of five items assessing a person’s satisfaction with their opportunities to participate in activities outside the home: *I visiting relatives and friends*, II *going on trips and traveling*, III *spending leisure time*, IV *meeting other people*, and V *living life as one wants*. Each item was scored from zero (very good) to four (very bad), and the total score was calculated by summing the responses for each question. The total score ranged from 0 to 20, with higher scores indicating more perceived limitations to autonomy in outdoor mobility. Cronbach’s alpha was 0.87 at baseline and 0.88 at follow-up. The IPA has previously demonstrated good construct validity, as well as internal reliability and test-retest reliability (Sibley et al., 2006).

### Covariates

Sociodemographic factors were included as covariates. Information on age and sex was obtained from the Digital and Population Data Services Agency, and for the analyses age cohorts was divided into two categories: 75-year or 80–85-year. Marital status was self-reported at baseline and divided into two categories: partnered or unpartnered. Perceived economic situation was also self-reported at baseline and divided into two categories: excellent/good or moderate/poor.

### Missing Data

There was no missing information on the main outcome variables at baseline, but a total of 23.6% of the sample would have been lost if we had used complete case analysis and excluded those who did not attend all phases of the follow-up. Therefore, full information maximum likelihood (FIML) was used to estimate missing information from those who attended at least some of the follow-up phases, resulting in a sample of 322 participants. In order to better meet the missing at random (MAR) condition, two auxiliary variables were used that correlated with the outcome variables and their missingness (Enders, 2022). The auxiliary variables employed were the baseline score on the 20-item Center for Epidemiologic Studies Depression Scale (CES-D; range 0–60, with higher scores indicating more depressive symptoms) (Radloff, 1977) and the baseline total score on the Short Physical Performance Battery test (SPPB; range 0–12, with higher scores indicating better lower extremity performance) (Guralnik et al., 1994). At follow-up, perceived autonomy in outdoor mobility was estimated for three (0.9%) participants, daily minutes of MVPA for 48 (14.9%) participants, distance walked in the 6MWT for 62 (19.3%) participants, and daily minutes of relative PA for 76 (23.6%) participants. To strengthen the robustness of our results, we also performed a sensitivity analysis using complete cases (*n* = 245), omitting observations with missing data.

### Statistical Analyses

Descriptive statistics (means and standard deviations (SD) for continuous variables, and percentages and frequencies for categorical variables) and Pearson correlation tests were derived using IBM SPSS Statistics (IBM Corp Released, 2021. IBM SPSS Statistics for Windows, version 28.0. Armonk, NY: IBM Corp). The Mplus statistical package, version 8.2 (Muthén & Muthén, 2017) was used for the cross-lagged panel analysis which examined the reciprocal associations between walking performance, PA, and perceived autonomy in outdoor mobility at two measurement points. Cross-lagged panel analysis allows the estimation of cross-lagged effects between variables (the effect of the previous variable on another variable measured on a later occasion), while controlling for autoregressive effects (the stability of the rank order of individuals from one occasion to the next) (Selig & Little, 2012). Parameters in the cross-lagged panel analysis were estimated using robust maximum likelihood estimation with robust standard errors (MLR), which corrects for bias in the case of non-normally distributed data. Given the skewed distribution of some variables, we also performed bootstrapping to ensure the robustness of our results. Missing information was estimated using FIML. The associations between all three dependent variables, that is, walking performance, relative PA or MVPA and autonomy in outdoor mobility, were examined simultaneously in the same model, but as the research interest was not in the associations between relative PA and MVPA, these were tested in separate models. Both relative PA and MVPA models were adjusted for the following baseline sociodemographic factors: sex, age, marital status, and perceived economic situation. Standardized estimation coefficients and robust standard errors (ES) are reported. Model fit was assessed using the following criteria: statistically non-significant (*p* > .05) chi-squared test (X^2^); root mean square error of approximation (RMSEA) values between 0.05 and 0.08; standardized root mean square residual (SRMR) values ≤0.08; the comparative fit index (CFI) values >0.95 (Schermelleh-En gel et al., 2003; Schreiber et al., 2006). The significance level was set at *p* < .05 for all analyses.

## Results

At baseline, the participants ranged in age from 74.7 to 85.3 years (mean age 78.1, SD 3.1) and 60.6% were women. Table 1 presents descriptive information on the study variables at baseline and follow-up, including the means and standard deviations, based on the observed data available at each time point.

**Table 1.** Descriptive Characteristics.

	Baseline 2017–2018		Follow-up 2021–22	
	*n*	% (*n*)	*n*	% (*n*)
Sex				
Female	322	60.6 (195)	322	60.6 (195)
Marital status				
Partnered	322	64.9 (209)	322	59.3 (191)
Perceived economic situation				
Excellent/good	322	63.4 (204)	322	70.2 (226)
	*n*	Mean (SD)	*n*	Mean (SD)
Age (years)	322	78.1 (3.2)	322	82.0 (3.2)
Height (m)	322	1.64 (0.09)	274	1.64 (0.09)
Weight (kg)	322	74.3 (13.0)	274	73.1 (12.6)
Wear time (d)	322	6.7 (0.7)	274	4.0 (0.1)
6MWT (m)	322	429.2 (76.5)	260	389.8 (75.0)
6MWT median MAD (g)	322	0.45 (0.12)	254	0.37 (0.10)
Relative PA (min/day)	322	8.2 (11.9)	246	12.1 (15.6)
Absolute MVPA (min/day)	322	49.7 (27.9)	274	40.5 (27.1)
Autonomy in outdoor mobility (score, range 0–20]	322	4.3 (3.4)	319	6.2 (4.2)

SD = standard deviation; 6MWT = 6-min walk test; MAD = mean amplitude deviation; relative PA = physical activity using acceleration corresponding to preferred walking intensity as a cut-point; MVPA = moderate-to-vigorous physical activity using acceleration corresponding to three METs as a cut-point.

Bivariate correlations showed weak to moderate correlations between higher MVPA, better walking performance and higher perceived autonomy in outdoor mobility (r = −0.141–0.475). Relative PA was only correlated with MVPA (r = 0.252–0.662), but not with walking performance or perceived autonomy in outdoor mobility (Supplementary Table 1S).

### Cross-Lagged Panel Model with Relative PA

**Figure 2.**
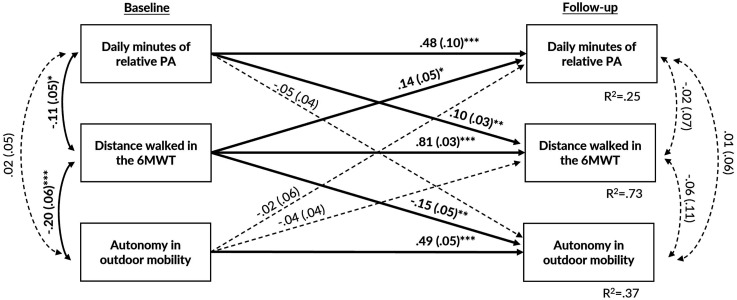
The standardized estimate coefficients (and standard errors) of the cross-lagged panel model between daily minutes of relative physical activity (PA), distance walked in the 6-min walk test (6MWT), and score of perceived autonomy in outdoor mobility. To simplify the model, covariates are not shown but include sex, age, marital status, and perceived economic situation. Statistically significant associations are bolded and presented with solid lines. The model fit: X^2^ (3) = 7.577, *p* = .056; RMSEA = 0.069; SRMR = 0.025; CFI = 0.990. Note. *: *p* < .05; **: *p* < .01; ***: *p* < .001.

Figure 2 shows the standardized estimation coefficients of the covariate-adjusted cross-lagged panel analysis with relative PA scaled to individuals’ preferred walking intensity, walking performance, and perceived autonomy in outdoor mobility. The autoregressive coefficients for relative PA, walking performance, and perceived autonomy in outdoor mobility showed relatively stable rank order over follow-up (*p* < .001). While controlling for covariates and baseline levels of autonomy in outdoor mobility and relative PA, better walking performance at baseline predicted higher levels of autonomy in outdoor mobility (β = −0.15, 95% CI = −0.24, −0.07) and higher levels of relative PA (β = 0.14, 95% CI = 0.03, 0.24) over 4 years of follow-up. In addition, while controlling for covariates and baseline walking performance and autonomy in outdoor mobility, higher levels of relative PA at baseline predicted better walking performance four years later (β = 0.10, 95% CI = 0.04, 0.15). Model fit indices showed adequate fit: X^2^ (3) = 7.577, *p* = .056; RMSEA = 0.069; SRMR = 0.025; CFI = 0.990.

### Cross-Lagged Panel Model with Absolute MVPA

**Figure 3.**
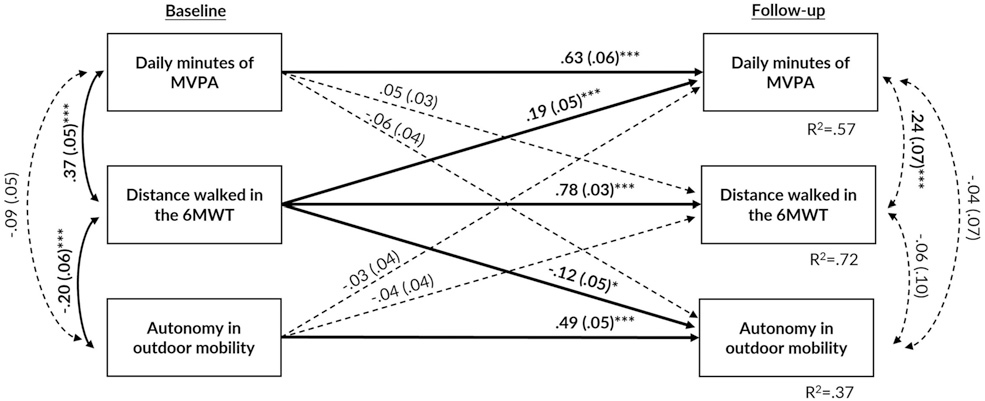
The standardized estimate coefficients (and standard errors) of the cross-lagged panel model between daily minutes of moderate-to-vigorous physical activity (MVPA), distance walked in the 6-min walk test (6MWT), and score of perceived autonomy in outdoor mobility. To simplify the model, covariates are not shown but include sex, age, marital status, and perceived economic situation. Statistically significant associations are bolded and presented with solid lines. The model fit: X^2^ (3) = 7.579, *p* = .056; RMSEA = 0.069; SRMR = 0.027; CFI = 0.994. Note. *: *p* < .05; **: *p* < .01; ***: *p* < .001.

Figure 3 presents the standardized estimation coefficients of the covariate-adjusted cross-lagged panel analysis with MVPA quantified with a fixed threshold, walking performance and perceived autonomy in outdoor mobility. As in the previous model, the autoregressive coefficients showed relatively strong stability over follow-up (*p* < .001). While controlling for covariates and baseline levels of autonomy in outdoor mobility and MVPA, better walking performance at baseline predicted higher autonomy in outdoor mobility (β = −0.12, 95% CI = −0.22, −0.04) and higher levels of MVPA (β = 0.19, 95% CI = 0.09, 0.27) over four years of follow-up. Neither MVPA nor perceived autonomy in outdoor mobility at baseline were associated with other outcome variables at follow-up. Model fit indices showed adequate fit: X^2^ (3) = 7.579, *p* = .056; RMSEA = 0.069; SRMR = 0.025; CFI = 0.994.

In sensitivity analyses with complete cases for both relative PA and MVPA models, only the cross-lagged association between walking performance and perceived autonomy in outdoor mobility was attenuated (Supplementary Table 2S and Figures 2S, 3S). The 95% confidence intervals (CIs) of the original model parameters and the bootstrapped 95% CIs showed consistency and supported robustness of our results, despite the skewed nature of some variables (Supplementary Figures 4S and 5S).

## Discussion

In this longitudinal study, we examined reciprocal associations between three dimensions of mobility, focusing on walking performance, relative and absolute PA, and perceived autonomy in outdoor mobility. Reciprocal associations between walking performance and PA were observed when the daily minutes of PA were quantified in relation to the person’s preferred usual walking intensity. However, reciprocal associations between walking performance and PA were not observed when the daily minutes of PA were assessed using a fixed threshold to quantify absolute MVPA. In both models, walking performance alone predicted perceived autonomy in outdoor mobility four years later.

Our results are partly consistent with previous studies. Based on self-reported PA, Metti et al. (2018) found reciprocal associations between physical capacity and PA, with physical capacity more consistently predicting declines in PA in both the short and long term. Another study, by Best et al. (2018), suggested that walking performance may have a greater influence on subsequent self-reported PA than vice versa. To our knowledge, only one study has investigated the reciprocal associations between accelerometer-based PA, quantified using fixed thresholds, and physical capacity in older adults (Yerrakalva et al., 2022). They found that better baseline physical capacity was associated with higher subsequent PA at all intensities. In addition, higher baseline MVPA was associated with faster subsequent walking speed and chair-stand speed, but not with better grip strength (Yerrakalva et al., 2022). We also found that baseline walking performance was associated with subsequent PA levels. For PA, our results showed that the association with later walking performance depended on how accelerometer-based PA was quantified. However, differences in PA assessment and analysis methods limit the direct comparability of our results with other studies.

Several factors may explain why reciprocal associations were observed between relative PA and walking performance, but not between walking performance and MVPA. The disparities in the associations may be due to variations in the actual intensities corresponding to the thresholds used to quantify relative PA and MVPA, as the intensity of the median acceleration during the preferred walking speed was observed to be higher than that corresponding to the three METs. A previous study has shown that older people may use compensatory actions, such as slowing their walking speed, to offset the increased energetic cost of walking associated with aging and chronic conditions (Schrack et al., 2012), which in turn contributes to a lower accelerometry threshold at 3 METs. Following the overload training principle (Garber et al., 2011; Visser et al., 2022), it is possible that the intensity of the threshold used to quantify MVPA was not sufficiently challenging for most participants. Conversely, engaging in PA exceeding one’s preferred walking intensity may better align with the intensity required to maintain physical function. This notion is also supported by a previous meta-analysis, which suggested that high-intensity exercise is pivotal for influencing habitual walking speed in older adults (Lopopolo et al., 2006). In addition to intensity, it is recognized that exercise adaptations are predominantly specific to the chosen modality, despite some overlapping effects (Izquierdo et al., 2021). In this sense, daily minutes of relative PA are most likely to have included brisk walking relative to a person’s physical capacity, whereas MVPA minutes may also have included other daily activities. However, future studies should continue to investigate the reciprocal associations between PA and walking performance, as well as other domains of physical capacity. In addition, the differential associations of relative and absolute accelerometer-based PA with physical capacity variables should be confirmed in future studies investigating older adults and other segments, such as those with chronic conditions that may alter physical capacity and modify walking performance.

In both relative PA and MVPA models, baseline walking performance was the sole predictor of subsequent perceived autonomy in outdoor mobility. Neither baseline relative PA nor MVPA predicted subsequent perceived autonomy in outdoor mobility. It has been suggested that autonomy in old age consists of both decisional and executive autonomy. The former refers to the ability of older adults to make choices and decisions based on their own preferences, whereas the latter pertains to the ability and freedom to act on the basis of decisional autonomy (Collopy, 1988). Presumably, the perceived autonomy in outdoor mobility is influenced by the opportunities to choose what to do, where to go, and who to meet. This, in turn, is influenced by walking performance, which facilitates or hinders the implementation of one’s preferences. In contrast, PA reflects actual behavior, which may not necessarily align with one’s desires, and can thus explain the lack of associations between PA and autonomy in outdoor mobility. Previous studies have also shown associations between better lower extremity performance and being non-frail with better perceived autonomy in outdoor mobility (Portegijs et al., 2014, 2016), supporting our findings that physical capacity contributes to perceived autonomy. Nevertheless, the attenuation of the cross-lagged association between walking performance and perceived autonomy in outdoor mobility observed in the complete case analyses warrants caution in interpreting the results. Additionally, in our previous study, we observed that the COVID-19 pandemic affected on older adults’ perceived autonomy in outdoor mobility despite their physical capacity (Lindeman et al., 2024). It is possible that the effects of the COVID-19 pandemic were particularly evident in decisional autonomy as a result of the reduction in opportunities for individuals to choose what to do and where to go. Consequently, the COVID-19 pandemic may have diminished the role of physical capacity and executive autonomy, which may further confound the observed associations between walking performance, PA, and autonomy in outdoor mobility.

This study has both strengths and limitations. The use of a cross-lagged panel analysis allowed us to investigate the longitudinal reciprocal associations between walking performance, PA, and autonomy in outdoor mobility, which to our best knowledge, has not been previously investigated. Furthermore, the use of accelerometers and the novel method to assess the role of PA independent of sex, age, and walking performance adds to the novelty of this study. Another strength of this study is the use of a relatively large population-based sample of community-dwelling older adults with four years of follow-up. Nevertheless, despite being a population-based sample, the participants who wore accelerometers had better health and physical function than those who declined free-living PA assessments (Portegijs et al., 2019). From a methodological point of view, the walk test may overestimate walking speed in the laboratory settings compared to free-living assessment (Higueras-Fresnillo et al., 2018). Therefore, it is possible that some participants may have walked at a faster pace during the 6MWT in the research laboratory than they typically do in free-living conditions, which may have resulted in an underestimation of the accumulated relative PA. However, walking intensity in terms of perceived exertion (on the Borg scale of 6 to 20) was on average moderate. It is also possible that the fixed threshold used to quantify MVPA was set too low for this study population due to the increased energetic cost of walking, resulting in an overestimation of MVPA minutes. Furthermore, the follow-up was conducted approximately two years after the onset of the COVID-19 pandemic, which may have influenced the observed associations, particularly in relation to perceived autonomy. In addition, as we used only the “autonomy outdoors” subscale of the IPA questionnaire and did not collect data on the other subscales of the IPA, the results may not be generalized to older people’s overall sense of participation and autonomy. Finally, although the cross-lagged panel analysis provides valuable insight into the prospective relationships between walking performance, PA, and autonomy in outdoor mobility, it should be noted that the conclusive causal relationship between them cannot be inferred due to the observational nature of the study.

## Conclusions

By accumulating more PA above one’s own preferred walking intensity, it is possible to maintain better walking performance, which, in turn, helps to maintain better perceived autonomy in outdoor mobility—an essential element of good quality of life. Our findings support early interventions in older adults that target walking performance and focus on PA intensities exceeding the preferred walking speed. Furthermore, we suggest that the use of individualized thresholds for MVPA in older adults would help to distinguish the effects of physical activity from physical capacity in predicting future changes in health and function.

## Supplemental Material

Supplemental Material - Reciprocal Associations Between Relative or Absolute Physical Activity, Walking Performance and Autonomy in Outdoor Mobility Among Older Adults: A 4-Year Follow-Up Supplemental Material for Reciprocal Associations Between Relative or Absolute Physical Activity, Walking Performance and Autonomy in Outdoor Mobility Among Older Adults: A 4-Year Follow-Up by Katja Lindeman, MSc, Kaisa Koivunen, PhD, Timo Rantalainen, PhD, Merja Rantakokko, PhD, Erja Portegijs, PhD, Taina Rantanen, PhD, and Laura Karavirta, PhD in Journal of Aging and Health

## Data Availability

The authors confirm that some access restrictions apply to the data. Researchers interested in using the data must obtain approval from the director of the AGNES study, Professor Taina Rantanen (taina.rantanen@jyu.fi), and are required to follow the protocol on the protection of privacy and comply with the relevant Finnish laws.
