# Supplementary material for: Reciprocal Associations Between Relative or Absolute Physical Activity, Walking Performance, and Autonomy in Outdoor Mobility Among Older Adults: A 4-Year Follow-Up
Source: J Aging Health. 2024 Sep 11;37(9):606–16. doi: 10.1177/08982643241282918 (PMC12405652; doi:10.1177/08982643241282918)
Supplement: Supplemental Material - Reciprocal Associations Between Relative or Absolute Physical Activity, Walking Performance and Autonomy in Outdoor Mobility Among Older Adults: A 4-Year Follow-Up [file sj-pdf-1-jah-10.1177_08982643241282918.pdf]

Supplementary materials

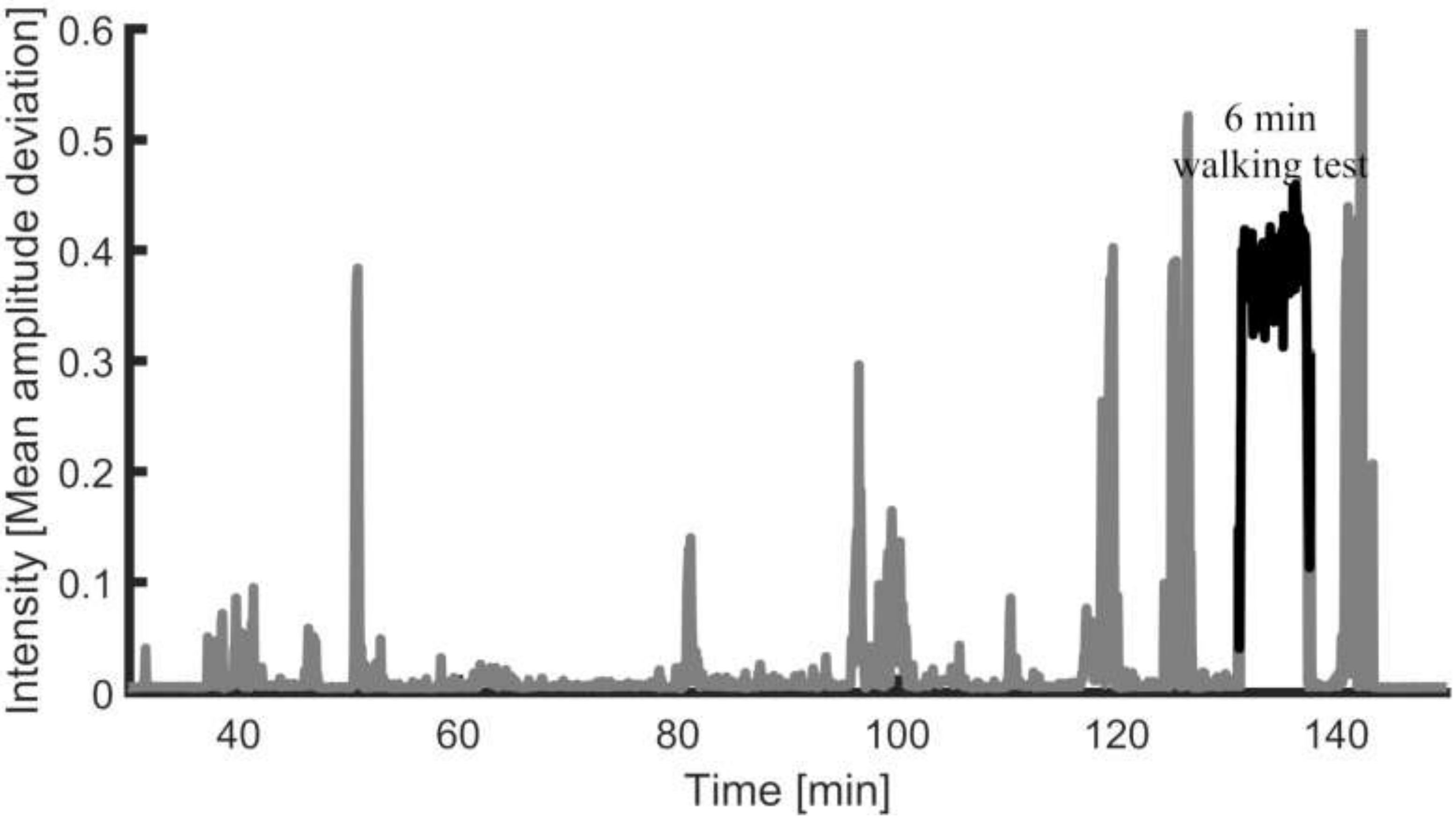

Supplementary Figure 1S. Visualization of the one participant's accelerometer recording during the visit to the research laboratory, including the 6-minute walk test (shown with a black line). The visualization was used to ensure that the 6-minute walk test was correctly identified from the accelerometer recording.

Supplementary Table 1S. Bi-variate correlations between moderate-to-vigorous physical activity (MVPA), relative physical activity (relative PA), distance walked in the 6-minute walk test (6MWT), and perceived autonomy in outdoor mobility (n = 322)

	**1**	**2**	**3**	**4**	**5**	**6**	**7**	**8**
1 MVPA, BL	1							
2 MVPA, FU	.721 ***	1						
3 Relative PA, BL	.454 ***	.252 ***	1					
4 Relative PA, FU	.424 ***	.662 ***	.483 ***	1				
5 6MWT, BL	.412 ***	.471 ***	-.085	.095	1			
6 6MWT, FU	.331 ***	.475 ***	-.001	.134 *	.816 ***	1		
7 Autonomy in outdoor mobility, BL	-.141 **	-.202 ***	-.002	-.071	-.275 ***	-.240 ***	1	
8 Autonomy in outdoor mobility, FU	-.212 ***	-.253 ***	-.045	-.083	-.334 ***	-.272 ***	.557 ***	1

MVPA = moderate-to-vigorous physical activity; relative PA = relative physical activity; 6MWT = 6-minute walk test; BL = baseline; FU = follow-up;
*** = p<0.001; ** = p<0.01; * = p<0.05.

Supplementary Table 2S. Descriptive characteristics of study sample with complete cases (n = 245)

	Baseline 2017-2018	Follow-up 2021-2022
	**% (n)**	**% (n)**
**Sex**		
Female	57.1 (140)	57.1 (140)
**Marital status**		
Partnered	65.9 (162)	61.4 (151)
**Perceived economic situation**		
Excellent / good	66.5 (163)	73.6 (181)
	**Mean (SD)**	**Mean (SD)**
**Age** [years]	77.8 (3.1)	81.7 (3.1)
**Height** [m]	1.65 (0.09)	1.65 (0.09)
**Weight** [kg]	74.8 (12.2)	72.9 (12.0)
**Wear time** [d]	6.7 (0.8)	4.0 (0.1)
**6MWT** [m]	440.0 (70.8)	389.3 (74.6)
**6MWT median MAD** [g]	0.46 (0.11)	0.37 (0.10)
**Relative PA** [min/day]	8.6 (12.5)	12.1 (15.6)
**Absolute MVPA** [min/day]	52.8 (27.4)	43.0 (26.8)
**Autonomy in outdoor mobility** [score, range 0-20]	4.0 (3.2)	5.4 (3.8)

SD = standard deviation; 6MWT = 6-minute walk test; MAD = mean amplitude deviation; relative PA = physical activity using acceleration corresponding to preferred walking intensity as a cut-point; MVPA = moderate-to-vigorous physical activity using acceleration corresponding to three METs as a cut-point.

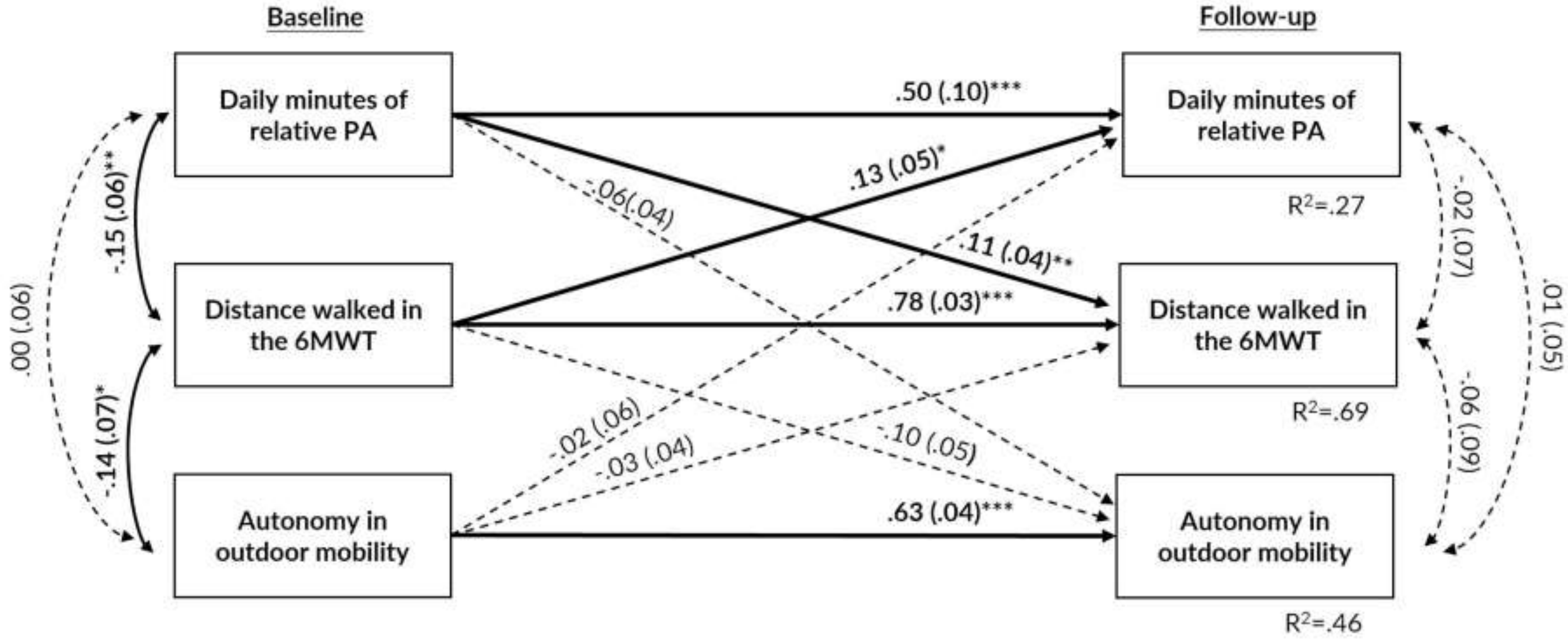

Supplementary Figure 2S. Cross-lagged panel model between daily minutes of relative physical activity (PA), distance walked in the 6-minute walk test (6MWT), and score of perceived autonomy in outdoor mobility with complete cases (n = 245). To simplify the model, covariates are not shown but include sex, age, marital status, and perceived economic situation. The standardized estimate coefficients (and standard errors) are presented. Statistically significant associations are bolded and presented with solid lines. The model fit: $X^2(0) = 0.000$, p = 0.000; RMSEA = 0.000; SRMR = 0.000; CFI = 1.000.

Note. *: p<.05; **: p<.01; ***: p<.001

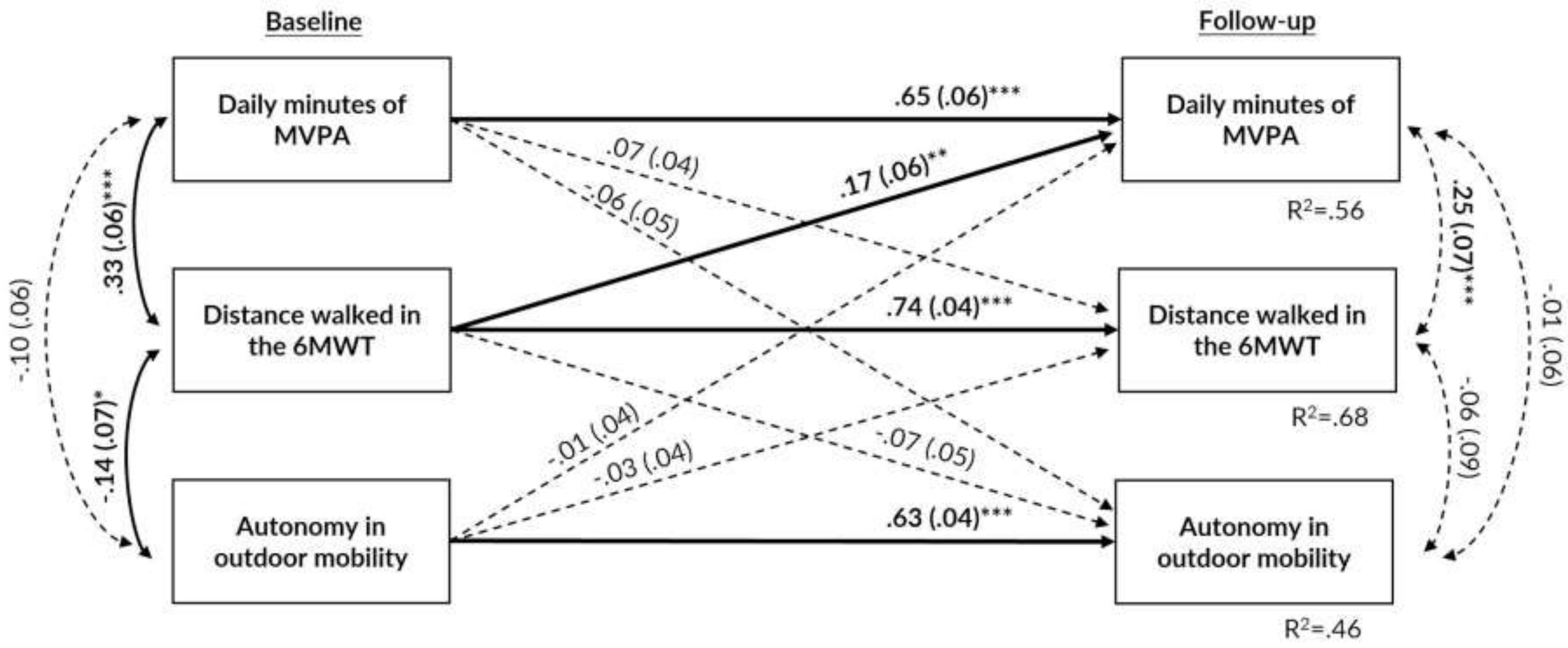

Supplementary Figure 3S. Cross-lagged panel model between daily minutes of moderate-to-vigorous physical activity (MVPA), distance walked in the 6-minute walk test (6MWT), and score of perceived autonomy in outdoor mobility with complete cases (n = 245). To simplify the model, covariates are not shown but include sex, age, marital status, and perceived economic situation. The standardized estimate coefficients (and standard errors) are presented. Statistically significant associations are bolded and presented with solid lines. The model fit: $X^2(0) = 0.000$, p = 0.000; RMSEA = 0.000; SRMR = 0.000; CFI = 1.000.

Note. *: p<.05; **: p<.01; ***: p<.001

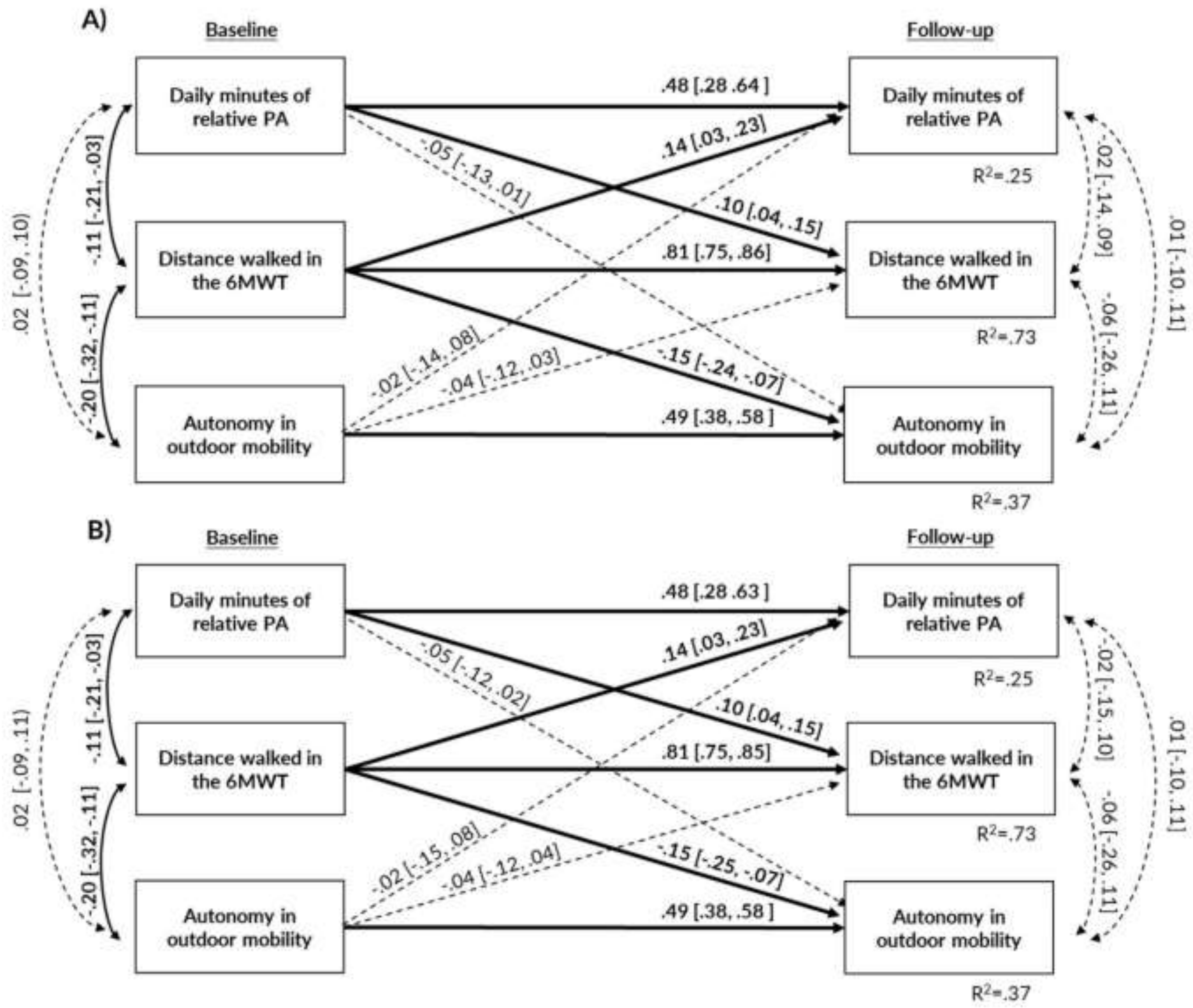

Supplementary Figure 4S. A) Cross-lagged panel model with the standardized estimate coefficients [and 95% CI] and B) bootstrapped cross-lagged panel model with the standardized estimate coefficients [and bootstrapped 95% CI]. To simplify the model, covariates are not shown but include sex, age, marital status. Statistically significant associations are bolded and presented with solid lines.

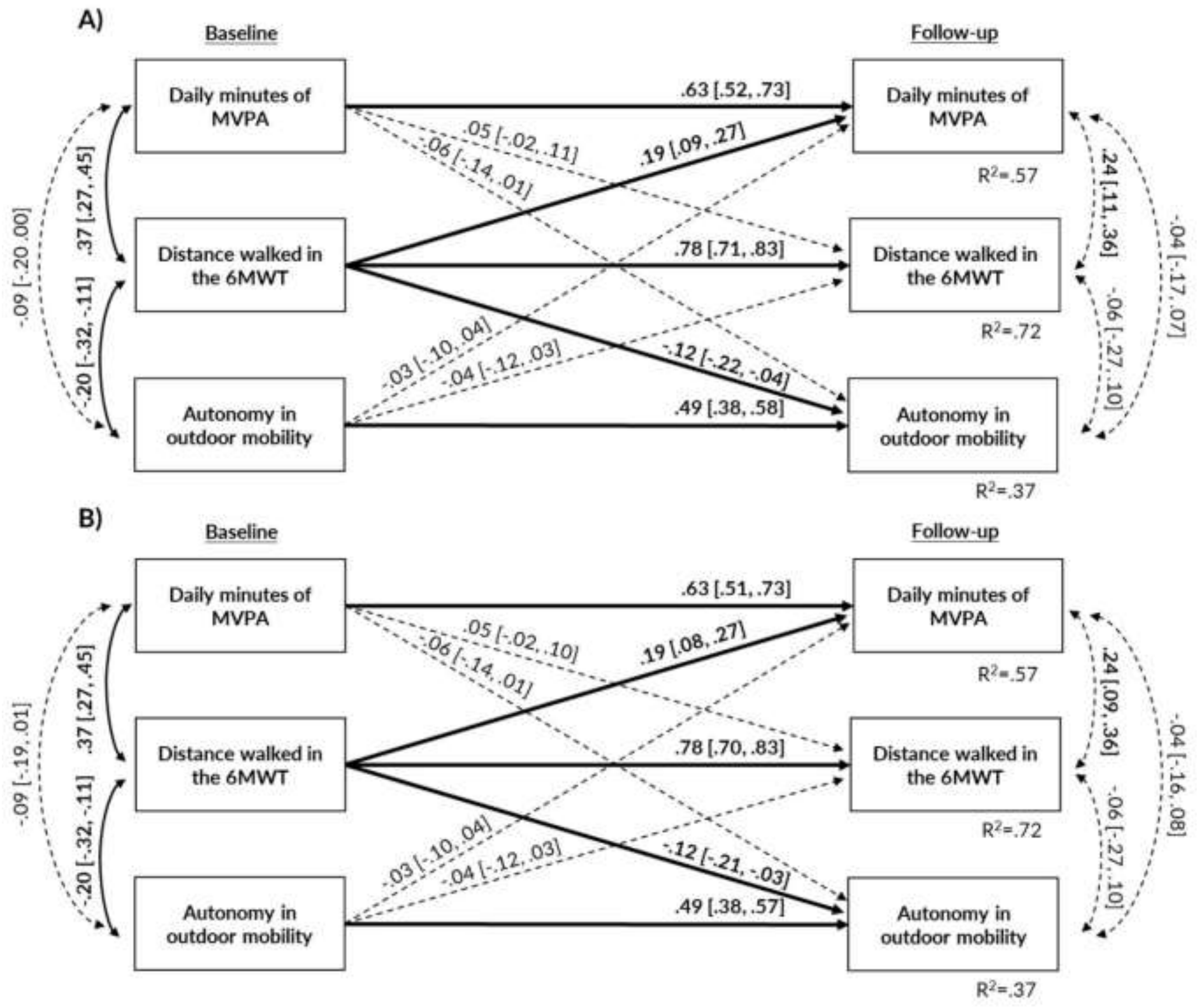

Supplementary Figure 5S. A) Cross-lagged panel model with the standardized estimate coefficients [and 95% CI] and B) bootstrapped cross-lagged panel model with the standardized estimate coefficients [and bootstrapped 95% CI]. To simplify the model, covariates are not shown but include sex, age, marital status. Statistically significant associations are bolded and presented with solid lines.
